# Catalytic enantioselective synthesis of selenium-containing atropisomers via C–Se bond formations

**DOI:** 10.3762/bjoc.21.186

**Published:** 2025-11-06

**Authors:** Qi-Sen Gao, Zheng-Wei Wei, Zhi-Min Chen

**Affiliations:** 1 State Key Laboratory of Synergistic Chem-Bio Synthesis, School of Chemistry and Chemical Engineering, Shanghai Key Laboratory for Molecular Engineering of Chiral Drugs, Shanghai Jiao Tong University, Shanghai 200240, P. R. China

**Keywords:** asymmetric catalysis, atropisomer, chiral selenium-containing compound, C–Se bond formation

## Abstract

Atropisomers are not only prevalent in biologically active natural products and pharmaceuticals, but they have also garnered increasing attention for their effectiveness as ligands and catalysts in the field of catalytic asymmetric synthesis. Asymmetric catalysis serves as a key strategy for the enantioselective synthesis of atropisomers, and significant progress has been made in recent years. However, selenium-containing atropisomers have long remained underexplored as synthetic targets, and only in recent years have they begun to attract increasing attention from the community. Recently, several synthetic approaches for constructing selenium-containing atropisomers have been reported, such as C–H selenylation of arenes, selenosulfonylation of vinylidene *o*-quinone methides (VQM), and hydroselenation of alkynes. Nevertheless, a comprehensive review that systematically summarizes these advances is currently lacking. This review aims to provide an overview of recent developments in the catalytic enantioselective synthesis of selenium-containing atropisomers via C–Se bond formation. We hope this review will serve as a valuable reference for researchers interested in further exploring this area.

## Introduction

**Figure 1:**
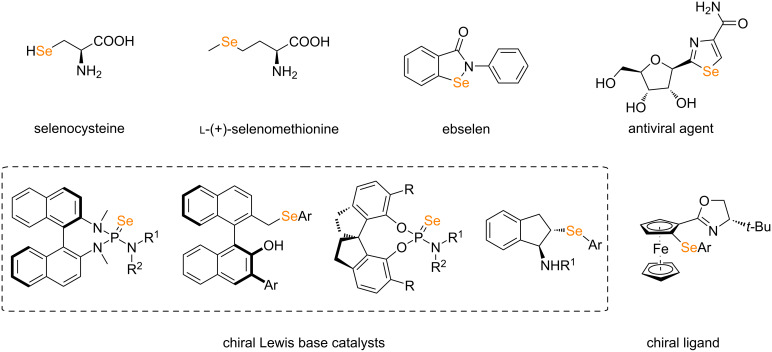
Representative examples of chiral selenium-containing compounds.

Selenium is an essential trace element for human body [1]. It plays an important role in metabolism. In 1817, the Swedish chemist Berzelius found that red residual mud was attached to the wall and bottom of the lead chamber when roasting pyrite to produce sulfuric acid. After analysis, it was confirmed that there was a new element in it. Referring to the name of tellurium (originally meaning earth), he named it selenium according to the word "Selene" in ancient Greek mythology [2]. Selenium is a non-metallic element, but compared with sulfur of the same main group, selenium has a larger atomic radius, smaller electronegativity, and exhibits certain metallic properties. Due to the special physical and chemical properties of selenium between metal and nonmetal, selenium not only has many applications in industry, but also plays an important role in many fields, such as electronics [3], agriculture [4], environmental protection [5] and cosmetics [6]. Chiral organic selenium-containing compounds also have important applications in organic synthesis and biomedicine [7–8]. These compounds can participate in asymmetric synthesis reactions and construct chiral molecules with specific stereoconfiguration, which is particularly critical for drug synthesis [9]. In the field of organic catalysis, chiral organic selenium-containing compounds can be used as chiral ligands or catalysts to participate in various types of asymmetric reactions, significantly improving the selectivity of reactions (Figure 1) [10–14].

Catalytic asymmetric synthesis is the main method to construct chiral organic selenium-containing compounds. Centrally chiral selenium-containing compounds can be efficiently constructed by catalytic asymmetric hydroselenation, catalytic asymmetric allyl substitution, catalytic asymmetric electrophilic selenylation/cyclization, etc. The research content of this part has already been covered by relevant reviews [15–17], so it is not within the scope of discussion in this review. Axially chiral selenium-containing compounds also play an irreplaceable role in asymmetric catalysis, functional materials, pharmaceutical chemistry and other fields. However, little attention has been paid to these compounds, which led to slow development and a relative lack of catalytic asymmetric synthesis methods. Only recently, methods for the formation of C–Se bonds have been established for the construction of selenium-containing atropisomers. However, there is no comprehensive review to summarize this great progress. In this paper, the catalytic asymmetric synthesis of axially chiral selenium-containing compounds by the formation of C–Se bonds is reviewed from three aspects.

## Review

### 1. Catalytic atroposelective synthesis of selenium-containing atropisomers by transition-metal-catalyzed C–H selenylation reactions

**Scheme 1:**
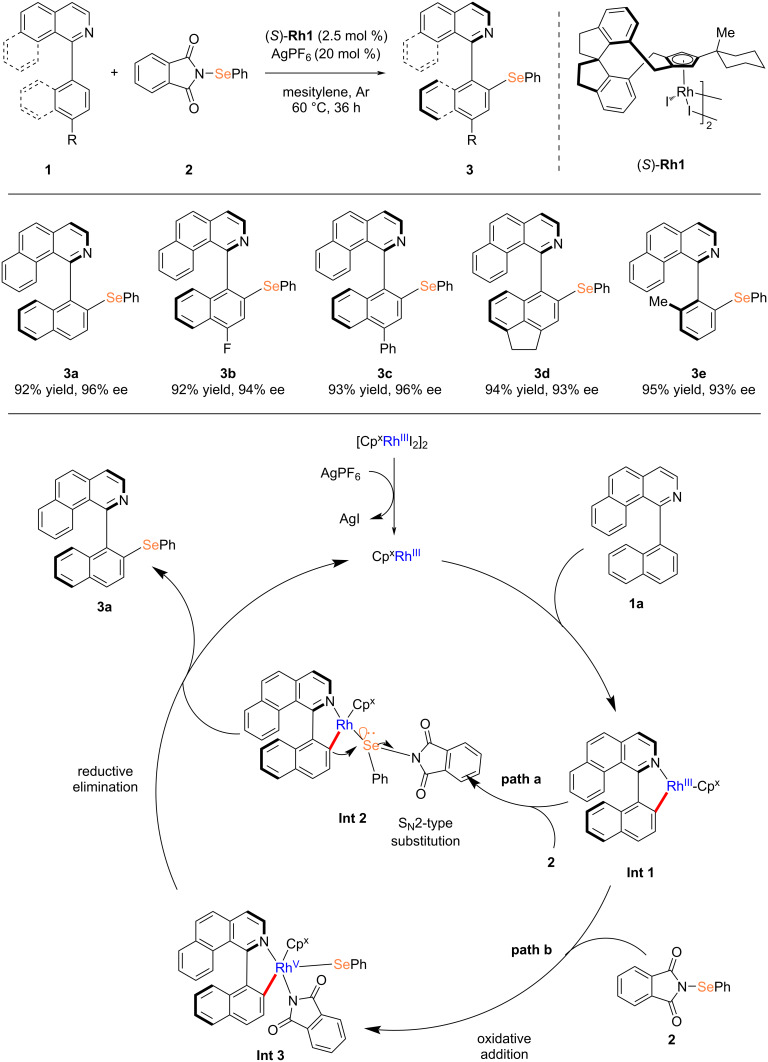
Rhodium-catalyzed atroposelective C–H selenylation reported by You’s group [18].

Transition-metal-catalyzed enantioselective C–H activation has emerged as a powerful strategy for the rapid synthesis of functionally enriched axially chiral diaryl compounds. However, due to the potential strong coordination between organoselenium compounds and transition metals, the direct construction of C–Se bonds via metal-catalyzed C–H bond functionalization remains a significant challenge. In 2024, You and co-workers reported a breakthrough in the enantioselective direct C–H selenylation of 1-arylisoquinolines and 2-(phenylselenyl)isoindoline-1,3-diones under rhodium catalysis to afford axially chiral diaryl selenides [18]. In this protocol, AgPF_6_ was employed as an additive and mesitylene served as the solvent. The reaction was conducted at 60 °C under an argon atmosphere. When 1-(naphthalen-1-yl)benzo[*h*]isoquinoline derivatives bearing various substituents were used as substrates, the reaction proceeded efficiently, yielding the products with excellent conversion rates (up to 95% yield) and high enantioselectivity (up to 96% ee). Notably, isoquinoline derivatives containing polycyclic naphthalene moieties or *ortho*-substituted phenyl groups also demonstrated good reactivity and compatibility. DFT calculations indicated that the C–Se bond formation proceeded through an S_N_2-type nucleophilic substitution mechanism (Scheme 1).

**Scheme 2:**
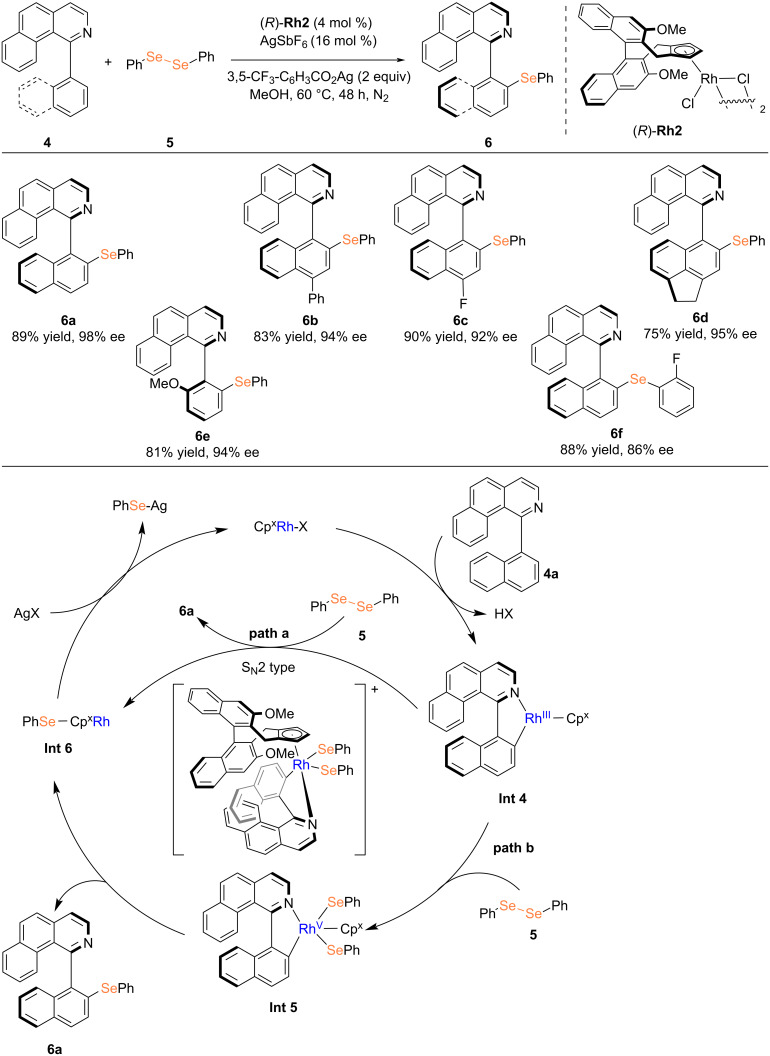
Rhodium-catalyzed atroposelective C–H selenylation reported by Li et al. [19].

In 2025, Li and co-workers reported a highly efficient rhodium-catalyzed enantioselective C–H selenylation reaction of 1-arylisoquinolines with diselenides, employing 3,5-(CF_3_)_2_C_6_H_3_CO₂Ag and AgSbF_6_ as additives [19]. When a *para*-fluorine substituent is present on the naphthalene ring of the substrate, the reaction proceeds with a yield of up to 90% and an enantioselectivity reaching 92% ee. The methodology demonstrates a broad substrate scope, accommodating various polycyclic naphthalene isoquinolines as well as phenyl-substituted benzoisoquinoline derivatives. Two plausible reaction mechanisms were proposed in the study: one involving oxidative addition of **Int 4**, a five-membered rhodium cyclic intermediate, followed by reductive elimination and the other proceeding via a bimolecular nucleophilic substitution pathway. In both pathways, the active chiral rhodium catalyst is regenerated through a silver salt-mediated recycling, with Ag–SePh being formed as a byproduct (Scheme 2).

### 2. Catalytic atroposelective synthesis of selenium-containing atropisomers by spontaneous selenosulfonylation of alkynes

**Scheme 3:**
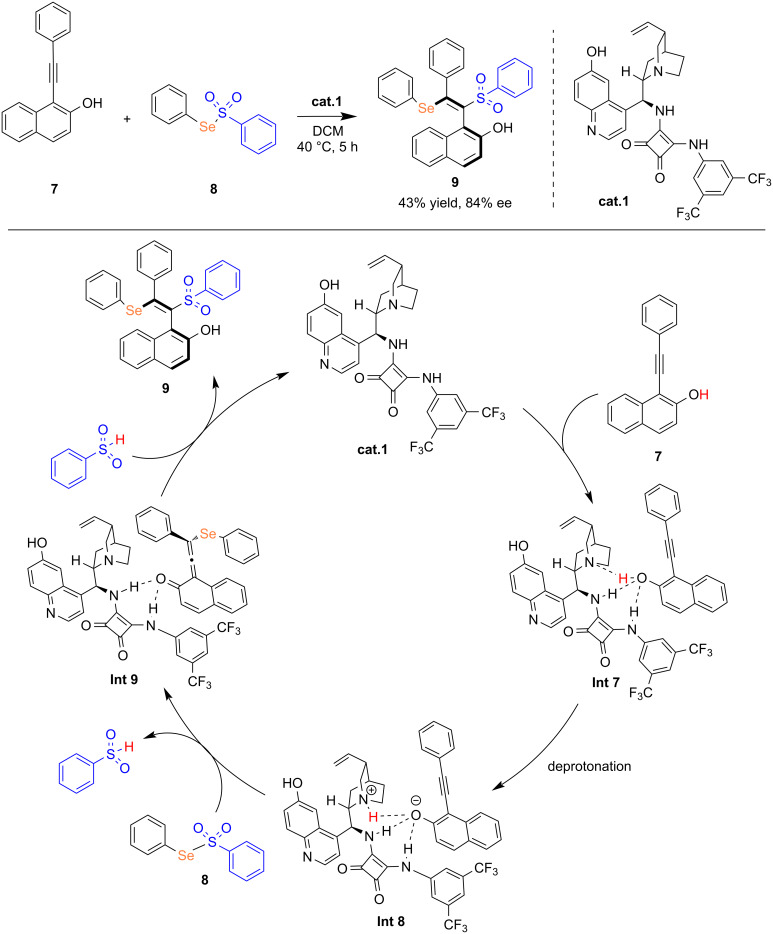
Organocatalytic asymmetric selenosulfonylation of alkynes.

Vinyl selenides, recognized as valuable synthetic intermediates and biologically active compounds, have been demonstrated to exhibit a broad spectrum of biological activities. Among them, the synthesis of β-(selenium)vinyl sulfones can be accomplished via selenosulfonylation reactions initiated by free radicals or cationic species. In 2019, Qin and co-workers reported a methodology enabling the difunctionalization of alkynes through selenosulfonylation of a VQM intermediate under mild reaction conditions [20]. This racemic transformation proceeds without the need for any catalyst or additive, and the reaction yielded the desired product at room temperature with high regioselectivity and stereoselectivity (*E*/*Z* ratio >99:1). Notably, when chiral catalyst (**cat.1**) was used, the reaction afforded the axially chiral product **9** in 43% yield with 84% ee. The proposed mechanism proceeds as follows. Catalyst **cat.1** initially engages substrate **7** through hydrogen bonding, forming intermediate **Int 7**. Subsequently, deprotonation of the naphthol group by quinuclidine yields intermediate **Int 8**. This intermediate then undergoes nucleophilic attack on the selenium atom in substrate **8**, leading to the formation of the VQM intermediate **Int 9** and benzenesulfonic acid. Finally, benzenesulfonic acid further reacts with the VQM intermediate to afford product **9**, concomitant with regeneration of the catalyst. This protocol provides a promising approach for the enantioselective synthesis of axially chiral styrenes containing both selenium and sulfone functionalities, highlighting the potential for further exploration of expanded catalyst and substrate scopes (Scheme 3).

### 3. Catalytic atroposelective synthesis of selenium-containing atropisomers by hydroselenation reactions of alkynes

**Scheme 4:**
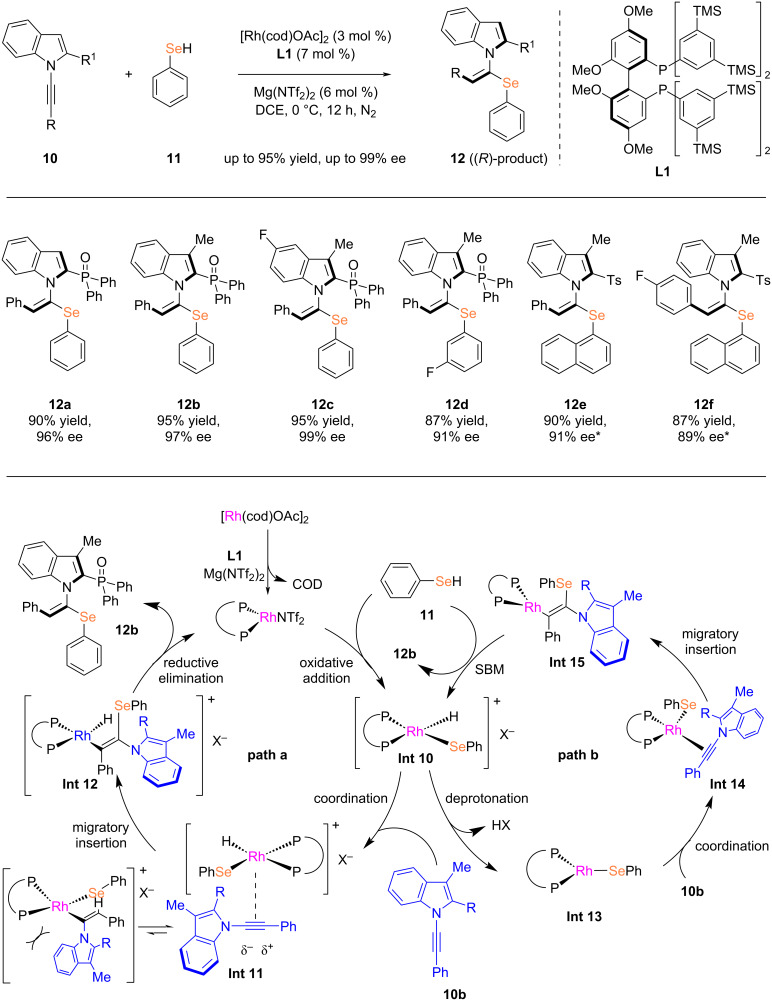
Rhodium-catalyzed asymmetric hydroselenation of 1-alkynylindoles. *DCE/DCM 2:1 (v/v), −50 °C.

The catalytic enantioselective hydroselenation of alkynes can provide an efficient and direct method for the synthesis of chiral vinyl selenides. However, to date, the enantioselective hydroselenation of alkynes remains underexplored. Building upon recent advances in rhodium-catalyzed asymmetric hydroselenation of olefins, in 2024, Li and co-workers reported an asymmetric hydroselenation reaction of 1-alkynylindoles using a catalytic system based on [Rh(cod)OAc]_2_ and Mg(NTf_2_)_2_ [21]. The Mg(II) salt not only activates the rhodium catalyst but also supplies the necessary NTf_2_^−^ anion for the reaction system, thereby significantly enhancing the catalytic performance. The developed catalytic system demonstrated high activity, excellent yields (mostly exceeding 85%), mild reaction conditions, broad functional group tolerance, as well as high regioselectivity, (*E*)-selectivity, and enantioselectivity (up to 99% ee). According to the kinetic study, the alkyne insertion step may be rate-limiting, as it involves the participation of selenol, alkyne, and the rhodium catalyst. The Rh(III) mechanism appears to be more plausible than route B, which can be attributed to the enhanced ion-pairing effect resulting from the higher oxidation state of rhodium (Scheme 4).

**Scheme 5:**
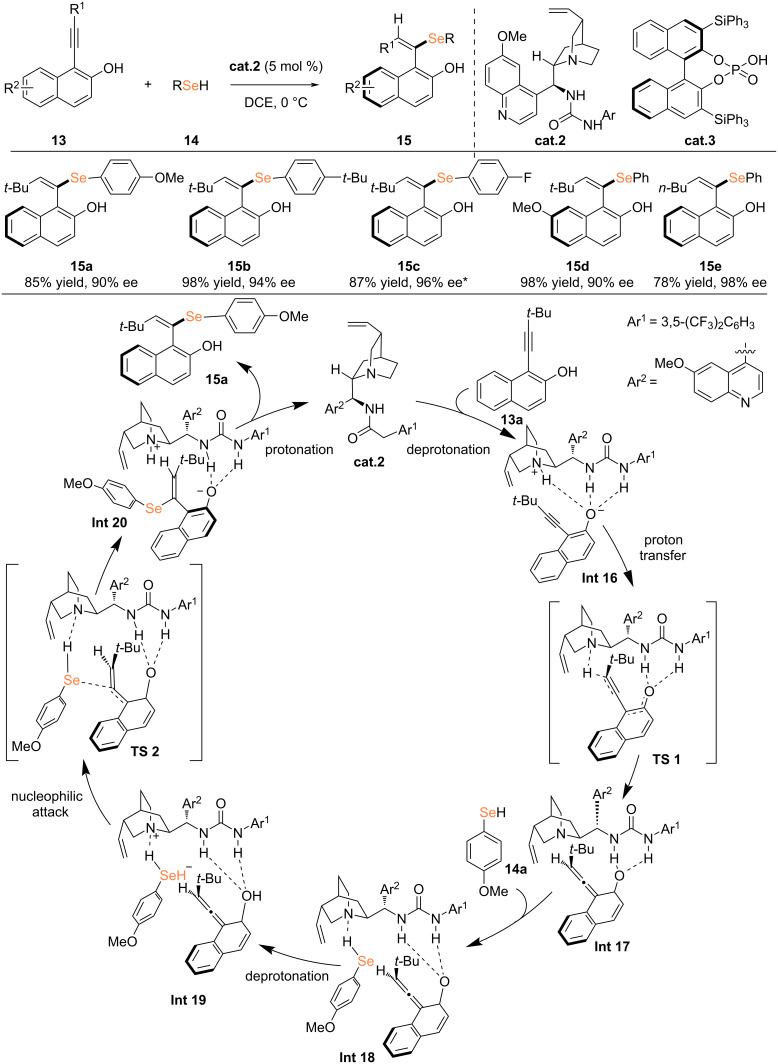
Organocatalytic atroposelective hydroselenation of alkynes. *Using **cat.3**, 4 h.

In 2025, Yang, Lu, and co-workers employed bifunctional catalysts, including chiral thiourea derivatives or chiral phosphoric acid, to activate alkynes and selenols through multiple hydrogen-bonding interactions, thereby achieving an enantioselective hydroselenation of alkynes [22]. All products demonstrated complete *E*-stereoselectivity (*E*/*Z* ratio >20:1). Notably, under the same reaction conditions, aliphatic selenols remained unreactive. Density functional theory (DFT) calculations revealed that the rate-determining step involves the nucleophilic attack of the selenium anion in intermediate **Int 16** on VQM to form intermediate **Int 17**. As bifunctional organic catalysts, chiral ureas can synergistically activate both alkynes and selenols, thereby addressing the challenge of overcoming the increased difficulty of racemization caused by the presence of bulky SeR groups (Scheme 5).

## Summary and Outlook

Overall, organic chemists have increasingly focused on the catalytic asymmetric synthesis of selenium-containing atropisomers, and significant progress has been made in recent years. Nevertheless, several limitations and challenges remain. For example, catalytic asymmetric electrophilic selenylation reactions have not yet enabled the effective synthesis of selenium-containing atropisomers. Moreover, the current methodologies are largely restricted to the use of selenium aryl groups. It is our intention to draw the attention of emerging researchers to this field and to promote its continued growth and development.

## Data Availability

Data sharing is not applicable as no new data was generated or analyzed in this study.
